# Identification of an Interaction between *VWF* rs7965413 and Platelet Count as a Novel Risk Marker for Metabolic Syndrome: An Extensive Search of Candidate Polymorphisms in a Case-Control Study

**DOI:** 10.1371/journal.pone.0117591

**Published:** 2015-02-03

**Authors:** Masahiro Nakatochi, Yasunori Ushida, Yoshinari Yasuda, Yasuko Yoshida, Shun Kawai, Ryuji Kato, Toru Nakashima, Masamitsu Iwata, Yachiyo Kuwatsuka, Masahiko Ando, Nobuyuki Hamajima, Takaaki Kondo, Hiroaki Oda, Mutsuharu Hayashi, Sawako Kato, Makoto Yamaguchi, Shoichi Maruyama, Seiichi Matsuo, Hiroyuki Honda

**Affiliations:** 1 Center for Advanced Medicine and Clinical Research, Nagoya University Hospital, Nagoya, Japan; 2 Department of Biotechnology, Graduate School of Engineering, Nagoya University, Nagoya, Japan; 3 Department of Nephrology, Nagoya University Graduate School of Medicine, Nagoya, Japan; 4 Department of CKD Initiatives, Nagoya University Graduate School of Medicine, Nagoya, Japan; 5 Innovative Research Center for Preventive Medical Engineering, Nagoya University, Nagoya, Japan; 6 Department of Basic Medicinal Sciences, Graduate School of Pharmaceutical Sciences, Nagoya University, Nagoya, Japan; 7 Safety & Health Promotion Division, Toyota Motor Corporation, Toyota, Japan; 8 Department of Healthcare Administration, Nagoya University Graduate School of Medicine, Nagoya, Japan; 9 Program in Radiological and Medical Laboratory Sciences, Nagoya University Graduate School of Medicine, Nagoya, Japan; 10 Department of Applied Molecular Biosciences, Graduate School of Bioagricultural Sciences, Nagoya University, Nagoya, Japan; 11 Department of Cardiology, Fujita Health University Second Hospital, Nagoya, Japan; IIBB-CSIC-IDIBAPS, SPAIN

## Abstract

Although many single nucleotide polymorphisms (SNPs) have been identified to be associated with metabolic syndrome (MetS), there was only a slight improvement in the ability to predict future MetS by the simply addition of SNPs to clinical risk markers. To improve the ability to predict future MetS, combinational effects, such as SNP—SNP interaction, SNP—environment interaction, and SNP—clinical parameter (SNP × CP) interaction should be also considered. We performed a case-control study to explore novel SNP × CP interactions as risk markers for MetS based on health check-up data of Japanese male employees. We selected 99 SNPs that were previously reported to be associated with MetS and components of MetS; subsequently, we genotyped these SNPs from 360 cases and 1983 control subjects. First, we performed logistic regression analyses to assess the association of each SNP with MetS. Of these SNPs, five SNPs were significantly associated with MetS (*P* < 0.05): *LRP2* rs2544390, rs1800592 between *UCP1* and *TBC1D9*, *APOA5* rs662799, *VWF* rs7965413, and rs1411766 between *MYO16* and *IRS2*. Furthermore, we performed multiple logistic regression analyses, including an SNP term, a CP term, and an SNP × CP interaction term for each CP and SNP that was significantly associated with MetS. We identified a novel SNP × CP interaction between rs7965413 and platelet count that was significantly associated with MetS [SNP term: odds ratio (OR) = 0.78, *P* = 0.004; SNP × CP interaction term: OR = 1.33, *P* = 0.001]. This association of the SNP × CP interaction with MetS remained nominally significant in multiple logistic regression analysis after adjustment for either the number of MetS components or MetS components excluding obesity. Our results reveal new insight into platelet count as a risk marker for MetS.

## Introduction

Metabolic syndrome (MetS) is characterized by a clustering of metabolic abnormalities, including central obesity, insulin resistance, dyslipidemia, and hypertension; moreover, it has been identified as a common precursor to the development of cardiovascular disease (CVD) [1]. The prevalence of MetS has been increasing in Japan during recent decades as a result of changes in diet and physical activity [2]. According to a survey by the Ministry of Health, Labor, and Welfare of Japan in 2007, one out of three males aged 30 to 59 years, who occupy the majority of Japanese employees, were strongly suspected of having or were likely to develop MetS [3]. National health insurers began conducting annual health check-ups of all customers between the ages of 40–74 in April 2008 [4]. There is an urgent need to find appropriate and sensitive risk markers to identify individuals at high risk for developing MetS and thereby prevent further increase in its incidence.

To explore risk markers for predicting MetS development, many studies have been performed utilizing health check-up data among different groups of people, such as company employees [5], people in hospitals [6,7], and members of various communities [8]. In these studies, many commonly measured clinical parameters (CPs) from routine health check-ups were reported to be associated with MetS. For example, Tao et al. reported that hematological parameters, such as white blood cell (WBC) count, low-density lipoprotein (LDL) cholesterol, and glutamic-pyruvic transaminase (GPT) were associated with MetS based on the health check-up data of a Beijing adult population [7]. These clinical parameters are expected to be clinical risk markers for MetS development.

In some studies, several combinations of clinical risk markers have also been explored based on health check-up data. We have previously shown that the combination of the γ-glutamyl transpeptidase (γ-GTP) level and WBC count was the most significant combinatorial risk marker associated with MetS based on the health check-up data of company employees [5]. We have also proposed the ratio of adiponectin to homeostasis model assessment of insulin resistance (A-H ratio) [8] as a combinational risk marker for MetS development based on community-based health check-up data. There is, however, a need to identify new combinational risk markers.

It is also known that genetic factors contribute to the development of MetS and MetS components (e.g., central obesity, insulin resistance, dyslipidemia, and hypertension). Recently, many single nucleotide polymorphisms (SNPs) that are associated with MetS and MetS components have been identified through candidate gene studies [9] and genome-wide association studies (GWAS) [10,11]. Although these SNPs were expected to be genetic risk markers for the development of MetS and MetS components, there are several problems that need to be resolved. First, most of the common variants, such as SNPs, confer relatively small increments in risk (1.1–1.5-fold) with regard to the development of common diseases, such as MetS and MetS components, and explain only a small proportion of heritability, which is the portion of phenotypic variance in a population that is attributable to additive genetic factors [12]. Additionally, there was only slight improvement in the ability to predict future MetS and MetS components by the simply addition of SNPs to clinical risk markers. For example, for predicting future type 2 diabetes, which is one of MetS components, the simply addition of 11 SNPs to clinical risk markers resulted in a slight increase in the area under the receiver operating-characteristic curve from 0.74 to 0.75 [13]. To improve the ability to predict future MetS and MetS components, combinational effects, such as SNP—SNP interaction, SNP—environment interaction, and SNP—clinical parameter (SNP × CP) interaction, should be also considered. Recently, some SNP × CP interactions have been reported as risk markers. For example, Manning et al. applied a joint meta-analysis approach to test associations with fasting glycemic traits and insulin resistance, which is thought to play a prominent role in MetS [14], on a genome-wide scale. Their results demonstrated that an interaction term between body-mass index (BMI) and an SNP that is located in an intergenic region between the *COBLL1* and *GRB14* is significantly associated with fasting insulin levels [15]. There is, however, a need to identify new SNP × CP interactions as risk markers for MetS development.

In this study, we performed a case-control study to explore novel SNP × CP interactions as risk markers for MetS based on health check-up data of Japanese male employees. We selected 99 candidate SNPs that were previously reported to be associated with MetS, MetS components, and coronary atherosclerosis. Subsequently, we screened SNPs that were significantly associated with MetS and explored SNP × CP interactions for association with MetS development. The explored interaction effect demonstrated in this study is expected to be utilized as a risk marker for MetS development. By combining conventional CP and SNP data, we can estimate the risk of future MetS development.

## Materials and Methods

### Study subjects

This study is case-control study for MetS, and part of an ongoing cohort, prospective observational study of MetS and chronic kidney disease (CKD). This original study has been following 33776 participants who underwent annual health check-ups for Toyota Motor Co., Ltd in both 2001 and 2009. Of these volunteers, 360 case subjects and 1983 control subjects who satisfied the definitions of cases or controls and attended health check-ups in 2011 or 2012 were randomly enrolled. Health check-up data were collected in 2001 and 2009, and DNA samples were obtained from case and control subjects in 2011 or 2012. This study was performed according to the guidelines of the Declaration of Helsinki. The study protocol was approved by Human Genome, Gene Analysis Research Ethics Committee of Nagoya University School of Medicine, and all participants provided written informed consent.

### Definitions of cases and controls

This study is a case-control study, and subjects who satisfied the definitions of cases or controls and attended health check-ups in 2011 or 2012 were randomly enrolled for this study. Health check-up data were collected in 2001 and 2009, and case / control groups were defined post hoc in 2009, while individuals meeting the criteria for MetS in 2001 were excluded. We used the criteria proposed by the Examination Committee of Criteria for the Metabolic Syndrome in Japan [16] to identify case and control subjects: 1) obesity, waist circumference ≥ 85 cm in 2009 or BMI ≥ 25 kg/m^2^ in 2001; 2) raised blood pressure, systolic blood pressure (SBP) ≥ 130 mmHg and/or diastolic blood pressure (DBP) ≥ 85 mmHg; 3) dyslipidemia, triglyceride ≥ 150 mg/dL and/or high-density lipoprotein (HDL)-cholesterol < 40 mg/dL; and 4) raised fasting blood sugar (FBS), FBS ≥ 110 mg/dL. Subjects were diagnosed with MetS if they were obese and showed any two of the other three criteria. Otherwise, subjects were classified as non-MetS. Then, we defined cases and controls according to the following criteria: cases, subjects who were classified as non-MetS in 2001 and were classified as MetS in 2009; controls, subjects who were classified as non-MetS in both 2001 and 2009.

### Measurements of CPs

The health examinations performed in 2001 and 2009 included physical measurements and serum biochemical measurements. Physical measurements of height, weight, and BMI were measured in the fasting state. Waist circumference was only measured in 2009. SBP and DBP were measured in the sitting position. Blood samples were obtained from subjects who had fasted for serum biochemical measurements. After the subject had rested for 10 min in the sitting position, 14 mL of blood was collected from the antecubital vein into tubes containing ethylenediaminetetraacetic acid (EDTA). After blood samples were sent to a clinical laboratory testing company, biochemical measurements were determined according to standard laboratory procedures. The study included the biochemical measurements of the following: (1) lipids: total cholesterol, triglyceride, and HDL-cholesterol; (2) carbohydrates: FBS; (3) hematology: red blood cell (RBC) count, white blood cell (WBC) count, hemoglobin (Hb), and platelet (PLT) count; (4) non-protein nitrogenous compounds: uric acid (UA); and (5) serum enzymes: aspartate aminotransferase (AST), alanine aminotransferase (ALT), and γ-glutamyl transpeptidase (γ-GTP).

### Selection of SNPs

Using public databases, such as PubMed and Online Mendelian Inheritance in Man, we selected 99 candidate SNPs that have been characterized and are associated with coronary atherosclerosis or vasospasm, obesity, hypertension, dyslipidemia, diabetes mellitus, hyperuricemia, or renal disease based on a comprehensive overview of vascular biology, coagulation and fibrinolysis cascades, platelet and leukocyte biology, as well as lipid and glucose metabolism and other metabolic factors (S1 Table).

### Genotyping SNPs

All SNPs were genotyped using the DigiTag2 assay [17] as previously described. Briefly, target fragments (including target SNP sites) are prepared by multiplex PCR from genomic DNA. A multiplexed oligonucleotide ligation assay was performed, and a labeling reaction was achieved with two 5′ query probes and one common probe prepared for a single SNP site. The 5′ query probes had a sequence complementary to the 5′-flanking region of the target SNP, and each of the probes had an allele-specific sequence. Two types of end digit (ED), CCGTGTCCACTCTAGAAAAACCT and ACCACCGCTTGAATACAAAACAT, were attached to each of the 5′ query probes. The 3′ query probes had a sequence complementary to the 3′-flanking region of the target SNP, and each of the probes had a first digit (D1) on its 3′ end. Next, a hybridization reaction with D1 probes on a DNA microarray (NGK Insulators, Ltd, Nagoya, Japan) was performed with separated areas. The genotyping success rate was > 99.8%. SNP rs1862513 was excluded from analysis because there was evidence of departure from the Hardy-Weinberg equilibrium (*P* < 0.05). Consequently, 98 SNPs remained for analysis. Primers, probe sequences, and PCR conditions for genotyping are shown in S2 Table and S3 Table.

### Study design

This study is a case-control study based on a prospective cohort data. The aim of this study is to identify interactions between SNPs and CPs from the 2001 data that successfully predicted MetS that was diagnosed in 2009. The explored interaction effect is expected to be utilized as a risk marker for MetS development. From the combined data of conventional CPs from 2001 and the SNPs, the risk of MetS development by 2009 could be estimated for each subject. To reduce false-positive interactions, we applied a two-step approach. We initially performed a screening analysis using the 98 SNPs. In this screening analysis, the cutoff *P* value was defined as less than 0.05 for logistic-regression analysis with adjustment for age. From the screening study, we selected five SNPs that were significantly associated with MetS. We then performed an interaction analysis to assess interactions between the five SNPs and 15 CPs that were measured in 2001 for predicting MetS that was diagnosed in 2009, including BMI, SBP, DBP, total cholesterol, HDL-cholesterol, triglyceride, FBS, RBC, WBC, Hb, PLT, UA, AST, ALT, and **γ**-GTP.

### Statistical analysis

The Hardy-Weinberg equilibrium was assessed using the Fisher’s exact test. Simple comparison of characteristics between case and control groups was carried out using the Mann—Whitney U test, Fisher’s exact test, and Student’s *t*-test. In the screening analysis, the associations between each SNP and MetS diagnosed in 2009 were assessed using logistic regression analysis with adjustment for age. We coded genotypes as 0, 1, or 2, depending on the number of copies of the minor alleles, for the multiple logistic regression analysis. In the interaction analysis, multiple logistic regression analyses, including an SNP term, a CP term, and an SNP × CP interaction term were performed for each combination of 15 clinical parameters and five SNPs that were statistically significant in the screening analysis. In the interaction analysis, the logistic regression models were fit as:

$$log(\frac{p_{case}}{1-p_{case}})=\beta_{0}+\beta_{Age}\times Age+\beta_{SNP}\times x_{SNP}+\beta_{CP}\times z_{CP}+\beta_{Interaction}\times (x_{SNP}-\mu_{SNP})\times z_{CP}$$ Model 1

$$log(\frac{p_{case}}{1-p_{case}})=\beta_{0}+\beta_{Age}\times Age+\beta_{SNP}\times x_{SNP}+\beta_{CP}\times z_{CP}+\beta_{Interaction}\times (x_{SNP}-\mu_{SNP})\times z_{CP}+\beta_{N_{Mets}}\times N_{Mets}$$ Model 2

$$log(\frac{p_{case}}{1-p_{case}})=\beta_{0}+\beta_{Age}\times Age+\beta_{SNP}\times x_{SNP}+\beta_{CP}\times z_{CP}+\beta_{Interaction}\times (x_{SNP}-\mu_{SNP})\times z_{CP}+\beta_{N_{Metsex}}\times N_{Metsex}$$ Model 3

where *p*_case_ is the probability that the subject is affected by MetS. *x*_*SNP*_ is the genotype coded as 0, 1, or 2 for each SNP. *μ*_*SNP*_ is the mean value of *x*_*SNP*_ for each SNP. *z*_CP_ is standardized value of each clinical parameter value. *N*_*MetS*_ is the number of MetS components. *N*_*MetSex*_ is the number of MetS components excluding obesity. Model 1 was used to explore interactions that are significantly associated with MetS. Models 2 and 3 were used to assess that the interaction was independent of the contribution of MetS components to MetS. Given that the distributions of HDL-cholesterol, triglyceride, RBC, WBC, Hb, PLT, UA, AST, ALT, and **γ**-GTP levels were skewed, these clinical parameter values were logarithmically transformed. To reduce the multi-collinearity, we centered genotypes for each SNP, *x*_*SNP*_, by subtracting the mean value from each genotype value, *μ*_*SNP*_, and standardized CP values to *z*_CP_ [18]. We transformed PLT count into a dichotomous value, based on median value of PLT count across all subjects (equal to 23.8×10^4^/μL): 1, equal to or greater than 23.8×10^4^/μL; and 0, less than 23.8×10^4^/μL. We compared the risk of MetS with regard to a combination of rs7965413 and dichotomous PLT count in an age-adjusted logistic regression model using the group with the CC genotype of rs7965413 and PLT count of < 23.8×10^4^/μL as a reference group. Linear regression analysis with adjustment for age was performed to assess the association of rs7965413 with PLT count in each case and control group. The heterogeneity of the regression coefficient between case and control groups was tested by the χ^2^-based Cochrane’s Q statistic. Statistical analysis was performed using R (www.r-project.org), PLINK [19], and METAL [20] softwares. In the interaction analysis, the significance level α was determined by dividing 0.05 by the number of CPs for Bonferroni correction (α = 0.05 / 15 = 0.0033). Otherwise, *P* < 0.05 was considered statistically significant.

## Results

### Characteristics of subjects

The characteristics of the study subjects in 2001 and 2009 are shown in Tables 1 and 2. There were significant differences among all characteristics between case and control groups in both 2001 and 2009. Of these characteristics, the number of MetS components and MetS components excluding obesity were the most significantly different between case and control groups in both 2001 and 2009.

**Table 1:** Characteristics of the study subjects for age and clinical parameters.

Characteristic	Case (n = 360 males)		Control (n = 1983 males)		*P* value (case vs control)	
	In 2001	In 2009	In 2001	In 2009	In 2001	In 2009
Age (years)	42 (37, 46)	50 (45, 54)	41 (33, 45)	49 (41, 53)	1.93 ×10^–9^	1.93 ×10^–9^
BMI (kg/m^2^)	24.6 (23.1, 26.5)	-	21.6 (20.0, 23.3)	-	1.28 ×10^–78^	-
Waist circumference (cm)	-	90.0 (87.0, 95.0)	-	79.0 (74.0, 83.0)	-	1.99 ×10^–160^
SBP (mmHg)	123 (114, 130)	133.5 (129, 138)	114 (107, 122)	116 (107, 123)	4.84 ×10^–34^	2.64 ×10^–113^
DBP(mmHg)	79 (72, 84)	86 (80, 89)	71 (65, 77)	73 (66, 78)	1.33 ×10^–37^	2.60 ×10^–93^
Total cholesterol (mg/dL)	205 (183, 229)	221 (196, 247)	184 (163, 208)	200 (180, 222)	2.17 ×10^–20^	9.43 ×10^–24^
HDL-cholesterol (mg/dL)	54 (45, 63)	49 (42, 58)	63 (54, 74)	60 (51, 71)	9.90 ×10^–30^	2.34 ×10^–40^
Triglyceride (mg/dL)	130 (92, 195)	185 (154, 238)	76 (56, 107)	81 (59, 114)	2.00 ×10^–65^	3.74 ×10^–128^
FBS (mg/dL)	95 (89, 102)	101 (91, 115)	90 (85, 96)	90 (85, 96)	9.79 ×10^–22^	7.72 ×10^–52^
RBC (×10^4^/μL)	491 (468, 513.3)	489 (465.8, 513)	476 (455, 499)	468 (446, 491)	4.86 ×10^–14^	2.70 ×10^–22^
WBC (×10^3^/μL)	7.0 (5.8, 8.2)	6.4 (5.5, 7.7)	6.0 (5.1, 7.2)	5.5 (4.7, 6.6)	2.42 ×10^–18^	1.59 ×10^–24^
Hb (g/dL)	15.5 (15.0, 16.2)	15.6 (14.9, 16.1)	15.1 (14.6, 15.7)	15 (14.3, 15.5)	5.44 ×10^–18^	1.21 ×10^–25^
PLT (×10^4^/μL)	25.0 (21.9, 28.9)	24.4 (21.0, 28.5)	23.6 (20.7, 27.1)	22.9 (20.0, 26.4)	2.02 ×10^–6^	1.25 ×10^–7^
UA (mg/dL)	6.3 (5.5, 7.2)	6.3 (5.5, 7.3)	5.8 (5.1, 6.5)	5.8 (5.0, 6.5)	1.92 ×10^–14^	3.11 ×10^–16^
AST (IU/L)	23 (19, 28)	24 (20, 31)	20 (17, 24)	20 (17, 23)	3.56 ×10^–13^	1.58 ×10^–34^
ALT (IU/L)	28 (20.8, 39)	31 (22, 47)	20 (15, 27)	18 (14, 25)	5.71 ×10^–33^	3.70 ×10^–62^
**γ**-GTP (IU/L)	42.5 (28, 69)	53 (35, 87)	26 (18, 42)	28 (20, 44)	1.05 ×10^–29^	2.40 ×10^–51^

Values are medians (1st quartile, 3rd quartile). The abbreviations of the characteristics: BMI, body mass index; SBP, systolic blood pressure; DBP, diastolic blood pressure; FBS, fasting blood sugar; RBC, red blood cell; WBC, white blood cell; Hb, hemoglobin; PLT, platelet; UA, uric acid; AST, aspartate aminotransferase; ALT, alanine aminotransferase; γ-GTP, gamma-glutamyl transpeptidase.

**Table 2:** Characteristics of the study subjects for MetS components.

Characteristic	Case (n = 360 males)		Control (n = 1983 males)		*P* value (case vs control)	
	In 2001	In 2009	In 2001	In 2009	In 2001	In 2009
MetS component						
Obesity, n (%)	157 (43.6)	360 (100)	188 (9.5)	239 (12.1)	4.61 ×10^–50^	< 1.00 ×10^–200^
Raised blood pressure, n (%)	128 (35.6)	326 (90.6)	236 (11.9)	264 (13.3)	3.12 ×10^–25^	3.30 ×10^–189^
Raised FBS, n (%)	33 (9.2)	131 (36.4)	45 (2.3)	58 (2.9)	5.35 ×10^–9^	7.60 ×10^–71^
Dyslipidemia, n (%)	151 (41.9)	306 (85.0)	192 (9.7)	197 (9.9)	2.17 ×10^–45^	3.45 ×10^–186^
Number of MetS components	1.3 ± 0.8	3.1 ± 0.3	0.3 ± 0.6	0.4 ± 0.7	4.10 ×10^–130^	< 1.00 ×10^–200^
Number of MetS components excluding obesity	0.9 ± 0.7	2.1 ± 0.3	0.2 ± 0.5	0.3 ± 0.5	5.10 ×10^–85^	< 1.00 ×10^–200^

Categorical data are n values (%). The numbers of MetS components are mean ± SD.

### Screening analysis

We initially assessed the association between MetS diagnosed in 2009 and 98 genotyped SNPs. Five out of 98 SNPs were found to be significantly associated with MetS (Table 3 and S4 Table), with *VWF* rs7965413 among those with the lowest *P* value [OR = 0.81, 95% confidence interval (CI) = 0.69–0.96; *P* = 0.012].

**Table 3:** Five SNPs that were nominally significantly associated with MetS in the screening study.

SNP	Chr	Position (GRCh37)	Near genes	Minor/major alleles	HWE *P* value	N		MAF		Logistic regression analysis	
						Case	Control	Case	Control	OR (95%CI)	*P* value
rs2544390	2	170,204,846	LRP2	C/T	0.231	360	1983	0.450	0.498	0.84 (0.71–0.98)	0.027
rs1800592	4	141,493,961	UCP1, TBC1D9	G/A	0.772	360	1982	0.456	0.502	0.83 (0.70–0.97)	0.022
rs662799	11	116,663,707	APOA5	G/A	0.515	360	1983	0.368	0.327	1.21 (1.03–1.43)	0.023
rs7965413	12	6,234,889	VWF	T/C	0.770	360	1980	0.400	0.452	0.81 (0.69–0.96)	0.012
rs1411766	13	110,252,160	MYO16, IRS2	T/C	0.515	360	1982	0.131	0.102	1.31 (1.03–1.67)	0.030

HWE, Hardy Weinberg equilibrium; MAF, minor allele frequency; OR, odds ratio; CI, confidence interval.

HWE *P* values were calculated by Fisher’s exact test.

OR and *P* values were calculated by multiple logistic regression analysis with adjustment for age.

OR value represents increased risk of MetS per minor allele copy in each SNP.

### Interaction analysis

Based on the results of the screening analysis, we focused on the five SNPs listed in Table 3 for further analysis. To explore SNP × CP interactions for MetS, we performed multiple logistic regression analyses including an SNP term, a CP term, and an SNP × CP interaction term. MetS was found to be significantly associated with an interaction between *VWF* rs7965413 and PLT in Model 1 (Table 4 and S5 Table). Furthermore, this association of the SNP × CP interaction with MetS remained nominally significant in multiple logistic regression analysis after adjustment for the number of MetS components in Model 2 and significant after adjustment for the number of MetS components excluding obesity in Model 3 (Table 4).

**Table 4:** Significant interaction effect between SNP rs7965413 and CP PLT in 2001 for MetS.

Model term	Model 1		Model 2		Model 3	
	OR (95%CI)	*P* value	OR (95%CI)	*P* value	OR (95%CI)	*P* value
SNP	0.78 (0.66–0.92)	0.004*	0.84 (0.70–1.02)	0.076	0.82 (0.68–0.98)	0.033
CP	1.35 (1.20–1.52)	1.17×10^–6^ ^†^	1.26 (1.10–1.44)	8.22×10^–4^ ^†^	1.31 (1.15–1.49)	4.14×10^–5^ ^†^
Interaction	1.33 (1.12–1.58)	0.001^†^	1.32 (1.08–1.60)	0.006*	1.35 (1.12–1.63)	0.002^†^

PLT, platelet; OR, odds ratio.

OR value for SNP term represents increased risk of MetS per minor allele T copy in rs7965413.

OR value for CP term represents increased risk of MetS per one standard deviation (SD) change in log_10_(PLT).

OR value for interaction term represents increased risk of MetS per one SD change in log_10_(PLT) × minor allele T copy in rs7965413.

* *P* < 0.05.

†*P* < 0.0033 (= 0.05/15)

**Fig 1:**
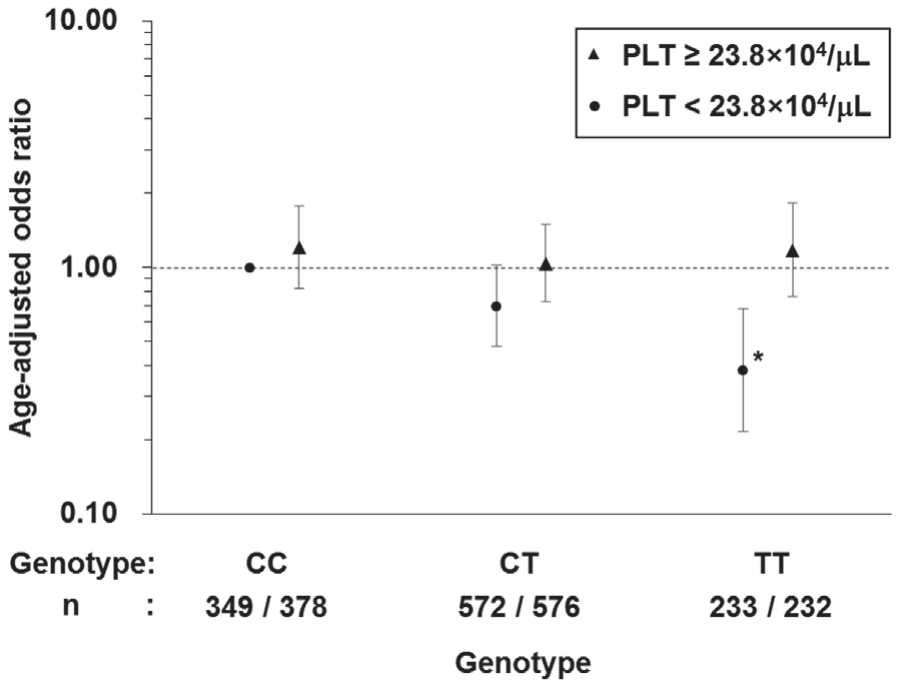
Age-adjusted ORs for MetS in platelet ≥ 23.8×10^4^/μL and < 23.8×10^4^/μL with different rs7965413 genotypes The vertical bars represent the 95% CIs. The horizontal dashed line indicates the null value (odds ratio (OR) = 1.0). OR represents risk of MetS development in the group with each genotype of rs7965413 and each dichotomous platelet count compared with the group with the CC genotype and PLT count of < 23.8×10^4^/μL. *: *P* < 0.05.

Furthermore, we transformed PLT count into a dichotomous value based on the median value of platelet count across all subjects, which was equal to 23.8×10^4^/μL and assessed an interaction effect between SNP rs7965413 and dichotomous PLT for MetS (Fig. 1). Multiple logistic regression analysis showed a significant interaction between SNP and dichotomous PLT for MetS (OR = 1.52, 95% CI = 1.09–2.12; *P* = 0.014). Among rs7965413 genotypes, OR was unchanged in subjects with PLT count ≥ 23.8×10^4^/μL, which is the median value of PLT count in the study participants. On the other hand, in subjects with PLT count < 23.8×10^4^/μL, OR decreased as the number of minor allele T increased.

### *VWF* rs7965413 and PLT count

Finally, we assessed the association of rs7965413 with PLT count in each group of cases and controls. In the case group, the minor allele T of rs7965413 was significantly positively associated with PLT. In the control group, the minor allele T was significantly negatively associated with PLT (Table 5). There was significant heterogeneity between case and control groups (*I* ^2^ = 90.2; *P* = 0.001).

**Table 5:** Associations of rs7965413 with PLT count in 2001.

Group	N	Linear regression analysis		Heterogeneity	
		*β* ± SE	P value	*I* ^2^	*P* value
Case	360	0.19 ± 0.08	0.013*	90.2	0.001*
Control	1980	-0.07 ± 0.03	0.021*		

PLT, platelet; *β*, partial regression coefficient; SE, standard error.

*β* value represents standard deviation change in standardized log_10_(PLT) per minor allele T change in rs7965413.

Multiple linear regression analysis was performed with adjustment for age.

* *P* < 0.05

## Discussion

We performed a case-control study of MetS based on the health check-up data of Japanese male employees and found significant associations of five SNPs with MetS, including *LRP2* rs2544390, rs1800592 between *UCP1* and *TBC1D9*, *APOA5* rs662799, *VWF* rs7965413, and rs1411766 between *MYO16* and *IRS2*. Furthermore, we identified a novel SNP × CP interaction for MetS, which was the interaction between *VWF* rs7965413 and platelet count. These SNPs and associated interaction are expected to be useful as risk markers for MetS development.

As shown in Tables 1 and 2, all characteristics, including CPs directly related to MetS, such as BMI, blood pressures, and cholesterols, as well as characteristics indirectly related to MetS, significantly differed between case and control groups. Of the CPs not directly related to MetS, levels of erythrocyte parameters, including RBC count, WBC count, hemoglobin, and PLT count in the case group were significantly higher than those in the control group. Several cross-sectional and longitudinal cohort studies have demonstrated that elevated erythrocyte parameters were associated with MetS [6,7,21]. Furthermore, Taniguchi et al., in their study on non-obese Japanese type 2 diabetes patients, found platelet count to be an independent predictor of insulin resistance [22]. Insulin resistance is thought to play a prominent role in MetS [14]. Our results were consistent with these reports.

As shown in Table 3, we found that *VWF* rs7965413 was significantly associated with MetS. *VWF* rs7965413 is located in the promoter region of the *VWF* gene. The *VWF* gene encodes von Willebrand factor (*v*WF). *v*WF promotes platelet adhesion and aggregation at sites of vascular injury, so it plays a prominent role in the formation of arterial thrombus [23]. Mutations in the *VWF* gene cause von Willebrand disease because of deficiency of *v*WF. It was also reported that *v*WF was associated with insulin level and insulin resistance [24]. For example, in a cross-sectional study, Meigs et al. reported that *v*WF antigen (*v*WF:Ag) level in men significantly increased across insulin quintiles [25]. Furthermore, *v*WF was reported to be associated with homeostasis model assessment—insulin resistance, which is an index of insulin resistance, and MetS [26]. Thus, an association between *v*WF plasma levels and CVD is expected and has been investigated. Several studies have reported that elevated *v*WF levels are positively associated with risk for coronary heart disease (CHD) [27,28]. Keightley et al. reported that the T allele (A allele in that study) of the *VWF* promoter variant rs7965413 had significantly lower *v*WF:Ag levels than the C allele (G allele in that study) [29] in a population of healthy individuals. In a case-control study, van der Meer et al. reported that a *VWF* promoter variant that was different from rs7965413 was also associated with risk for CHD [30]. However, because other studies could not detect an association with CHD [31], it is unclear if *VWF* promoter variants contribute significantly to CVD risk. We found that risk of MetS decreased as the number of T alleles of rs7965413 increased. This result may indicate a causal relationship between rs7965413 and CVD that is mediated via *v*WF:Ag and MetS.

As shown in Table 4, we identified a novel SNP × CP interaction between rs7965413 and platelet count significantly associated with MetS. This association of the SNP × CP interaction with MetS remained nominally significant in multiple logistic regression analysis after adjustment for the number of MetS components and significant after adjustment for the number of MetS components excluding obesity. This result indicates that this SNP × CP interaction is an independent risk marker for MetS. As described above, rs7965413 contributes to *v*WF:Ag levels, and *v*WF promotes platelet adhesion. The statistical interaction term between rs7965413 and platelet count is expected to reflect the biological interaction between *v*WF and PLT. This result reveals new information regarding platelet count as a risk marker for MetS. Moreover, for rs7965413, frequencies of the T allele across different ethnicities were as follows: African, 0.372; American, 0.445; Asian, 0.427; European, 0.621 in the 1000 Genomes Project Phase I version 3. This SNP was observed across different races, so the interaction may also be observed in other race/ethnic groups.

We found the association of rs7965413 with PLT count in both case and control groups. A GWAS recently showed that an SNP upstream of *VWF*, rs7342306, was associated with platelet count [32]. Although we did not genotype rs7342306, there was weak linkage disequilibrium (LD) between this SNP and rs7965413 in the 1000 Genomes Project Phase I version 3 ASN (r^2^ = 0.123, *D*’ = 0.401). Thus, the contribution of rs7965413 to platelet count is expected to be independent of the contribution of rs7342306 to platelet count. Our results indicate significant heterogeneity between case and control groups. The association of rs7965413 with platelet count might not be detected in the GWAS because of this heterogeneity.

It was also reported that variants in the *VWF* gene were associated with traits related to blood pressure, which was one of MetS components. Ruixing et al. reported that *VWF* rs1063856 (Thr789Ala) was significantly associated with hypertension in women [33]. Defago et al. reported that *VWF* rs2239153 was significantly associated with salt sensitivity [34]. These variants were weak LD with rs7965413 in the 1000 Genomes Project Phase I version 3 ASN (r^2^ = 0.0, *D*’ = 0.013 for rs1063856; r^2^ = 0.066, *D*’ = 0.288 for rs2239153). Thus, the contribution of rs7965413 to MetS is expected to be independent of the contribution of these variants to blood pressure.

As shown in Table 3, we found that four other SNPs were significantly associated with MetS: *LRP2* rs2544390, rs1800592 between *UCP1* and *TBC1D9*, *APOA5* rs662799, and rs1411766 between *MYO16* and *IRS2*. Of these SNPs, *APOA5* rs662799 was frequently reported to be associated with MetS and dyslipidemia, which was one of MetS components, based on several populations, including Japanese [35], Chinese [36], and Caucasian [37] populations. Our results are consistent with these reports. SNP rs1800592 between *UCP1* and *TBC1D9* is an A→G point mutation at the—3826 position in the 50 flanking region of the *UCP1* gene. The *UCP1* gene is a candidate gene for obesity and type 2 diabetes mellitus because the gene has been found to decrease mitochondrial membrane potential and increase thermogenesis [38]. Many association studies were conducted in various populations to elucidate the association of rs1800592 with obesity phenotypes, diabetes mellitus, and lipid/lipoprotein-related disease, but the results have been controversial [39,40]. Our results indicate a significant association of this SNP with MetS. However, the associations of this SNP with obesity, diabetes, and lipids were not replicated in our data (results not shown). *LRP2* rs2544390 is located in the *LRP2* gene. It has been reported that the T allele of this SNP is associated with higher serum UA [41]. Our results indicate that the T allele was associated with risk of MetS development. However, the association of this SNP with UA was not replicated in our data (results not shown). SNP rs1411766 localizes to an intergenic region ~384 kb telomeric to *MYO16* and 120 kb centromeric to *IRS2*. This SNP was reported increase susceptible to diabetic nephropathy, as determined by a GWAS in European-American subjects with type 1 diabetes [42] and was also observed to be associated with susceptibility to diabetic nephropathy in a Japanese population with type 2 diabetes [43]. Obesity, hypertension, and other MetS components are expected to either cause or exacerbate the progression of nephropathy, independent of diabetes [44]. The rs1411766 was both a risk allele for diabetic nephrology as well as MetS development in our study. Although the function of rs1411766 has not been understood, this SNP may contribute to the development of MetS components excluding diabetes and result in the development of diabetic nephropathy.

Our study has some limitations. First, this is a case-control and exploratory study that does not establish a cause-and-effect relationship. Future studies are thus necessary to evaluate the predictive potential of SNP × CP interactions as risk markers in prospective cohorts. We assumed that the interaction is linear; that is, the per-allele effect of an SNP changes across the continuous spectrum of a CP. However, if the interaction effect is nonlinear or a threshold effect exists, in which case the association would only be present in one extreme of the CP distribution, this analysis is not suitable and other analytical methods should be applied.

In conclusion, our data demonstrate associations of five SNPs with MetS and of an interaction between SNP rs7965413 and platelet count for MetS. Our results reveal new insight into PLT count as a risk marker for MetS.

## Supporting Information

S1 Table The 99 genotyped SNPs. (DOCX)

S2 Table Multiplex PCR primers and PCR conditions for genotyping the 99 SNPs. (XLSX)

S3 Table Probes for genotyping the 99 SNPs. (XLSX)

S4 Table Multiple logistic regression analysis of the association between 98 SNPs and MetS in a screening analysis. (XLSX)

S5 Table Multiple logistic regression analysis including interactions between five SNPs and 15 clinical parameters in 2001 for MetS. (XLSX)

## References

[1] Gami AS, Witt BJ, Howard DE, Erwin PJ, Gami LA, et al (2007) Metabolic syndrome and risk of incident cardiovascular events and death: a systematic review and meta-analysis of longitudinal studies. J Am Coll Cardiol 49: 403–414. 17258085 10.1016/j.jacc.2006.09.032

[2] McCurry J (2004) Japanese people warned to curb unhealthy lifestyles. Health experts urge a return to dietary basics to prevent future health problems. Lancet 363: 1126 15068019 10.1016/s0140-6736(04)15942-4

[3] Ministry of Health, Labour and Welfare of Japan (2007) Outline for the results of the national health and nutrition survey Japan, 2007 Available: http://www0.nih.go.jp/eiken/english/research/pdf/nhns2007.pdf. Accessed 4 July 2014. 10.1093/jxb/erm028 25506957

[4] Ministry of Health, Labour and Welfare of Japan (2007) Chapter 4: Future health promotion and medicine—reforming the medical architecture In: The white paper of the Ministry of Health and Welfare in Heisei 19. In: Kyokai Kousei Toukei, editor. Tokyo pp. 97–161.

[5] Ushida Y, Kato R, Niwa K, Tanimura D, Izawa H, et al (2012) Combinational risk factors of metabolic syndrome identified by fuzzy neural network analysis of health-check data. BMC Med Inform Decis Mak 12: 80 10.1186/1472-6947-12-80 22853735 PMC3469424

[6] Wu S, Lin H, Zhang C, Zhang Q, Zhang D, et al (2013) Association between erythrocyte parameters and metabolic syndrome in urban Han Chinese: a longitudinal cohort study. BMC Public Health 13: 989 10.1186/1471-2458-13-989 24144016 PMC4016498

[7] Tao LX, Li X, Zhu HP, Huo D, Zhou T, et al (2013) Association of hematological parameters with metabolic syndrome in Beijing adult population: a longitudinal study. Endocrine. 10.1007/s12020-013-0144-3 24091543

[8] Nakatochi M, Miyata S, Tanimura D, Izawa H, Asano H, et al (2011) The ratio of adiponectin to homeostasis model assessment of insulin resistance is a powerful index of each component of metabolic syndrome in an aged Japanese population: results from the KING Study. Diabetes Res Clin Pract 92: e61–65. 10.1016/j.diabres.2011.02.029 21458098

[9] Povel CM, Boer JM, Reiling E, Feskens EJ (2011) Genetic variants and the metabolic syndrome: a systematic review. Obes Rev 12: 952–967. 10.1111/j.1467-789X.2011.00907.x 21749608

[10] Kristiansson K, Perola M, Tikkanen E, Kettunen J, Surakka I, et al (2012) Genome-wide screen for metabolic syndrome susceptibility Loci reveals strong lipid gene contribution but no evidence for common genetic basis for clustering of metabolic syndrome traits. Circ Cardiovasc Genet 5: 242–249. 10.1161/CIRCGENETICS.111.961482 22399527 PMC3378651

[11] Zabaneh D, Balding DJ (2010) A genome-wide association study of the metabolic syndrome in Indian Asian men. PLoS One 5: e11961 10.1371/journal.pone.0011961 20694148 PMC2915922

[12] Hindorff LA, Sethupathy P, Junkins HA, Ramos EM, Mehta JP, et al (2009) Potential etiologic and functional implications of genome-wide association loci for human diseases and traits. Proc Natl Acad Sci U S A 106: 9362–9367. 10.1073/pnas.0903103106 19474294 PMC2687147

[13] Lyssenko V, Jonsson A, Almgren P, Pulizzi N, Isomaa B, et al (2008) Clinical risk factors, DNA variants, and the development of type 2 diabetes. N Engl J Med 359: 2220–2232. 10.1056/NEJMoa0801869 19020324

[14] Weyer C, Funahashi T, Tanaka S, Hotta K, Matsuzawa Y, et al (2001) Hypoadiponectinemia in obesity and type 2 diabetes: close association with insulin resistance and hyperinsulinemia. J Clin Endocrinol Metab 86: 1930–1935. 11344187 10.1210/jcem.86.5.7463

[15] Manning AK, Hivert MF, Scott RA, Grimsby JL, Bouatia-Naji N, et al (2012) A genome-wide approach accounting for body mass index identifies genetic variants influencing fasting glycemic traits and insulin resistance. Nat Genet 44: 659–669. 10.1038/ng.2274 22581228 PMC3613127

[16] Examination Committee of Criteria for the Metabolic Syndrome in Japan (2005) Metabolic syndrome—definition and diagnostic criteria in Japan. J Jpn Soc Int Med 94: 188–203. (in Japanese)

[17] Nishida N, Tanabe T, Takasu M, Suyama A, Tokunaga K (2007) Further development of multiplex single nucleotide polymorphism typing method, the DigiTag2 assay. Anal Biochem 364: 78–85. 17359929 10.1016/j.ab.2007.02.005

[18] Aiken LS, West SG, Reno RR (1991) Multiple regression: testing and interpreting interactions. Newbury Park, Calif.: Sage Publications xi, 212 p.

[19] Purcell S, Neale B, Todd-Brown K, Thomas L, Ferreira MA, et al (2007) PLINK: a tool set for whole-genome association and population-based linkage analyses. Am J Hum Genet 81: 559–575. 17701901 10.1086/519795 PMC1950838

[20] Willer CJ, Li Y, Abecasis GR (2010) METAL: fast and efficient meta-analysis of genomewide association scans. Bioinformatics 26: 2190–2191. 10.1093/bioinformatics/btq340 20616382 PMC2922887

[21] Nebeck K, Gelaye B, Lemma S, Berhane Y, Bekele T, et al (2012) Hematological parameters and metabolic syndrome: findings from an occupational cohort in Ethiopia. Diabetes Metab Syndr 6: 22–27. 10.1016/j.dsx.2012.05.009 23014250 PMC3460271

[22] Taniguchi A, Fukushima M, Seino Y, Sakai M, Yoshii S, et al (2003) Platelet count is independently associated with insulin resistance in non-obese Japanese type 2 diabetic patients. Metabolism 52: 1246–1249. 14564674 10.1016/s0026-0495(03)00099-4

[23] Ruggeri ZM, Ware J (1993) von Willebrand factor. FASEB J 7: 308–316. 8440408 10.1096/fasebj.7.2.8440408

[24] Vischer UM (2006) von Willebrand factor, endothelial dysfunction, and cardiovascular disease. J Thromb Haemost 4: 1186–1193. 16706957 10.1111/j.1538-7836.2006.01949.x

[25] Meigs JB, Mittleman MA, Nathan DM, Tofler GH, Singer DE, et al (2000) Hyperinsulinemia, hyperglycemia, and impaired hemostasis: the Framingham Offspring Study. JAMA 283: 221–228. 10634338 10.1001/jama.283.2.221

[26] Ragab A, Abousamra NK, Higazy A, Saleh O (2008) Relationship between insulin resistance and some coagulation and fibrinolytic parameters in patients with metabolic syndrome. Lab Hematol 14: 1–6. 10.1532/LH96.07017 18403313

[27] Folsom AR, Wu KK, Rosamond WD, Sharrett AR, Chambless LE (1997) Prospective study of hemostatic factors and incidence of coronary heart disease: the Atherosclerosis Risk in Communities (ARIC) Study. Circulation 96: 1102–1108. 9286936 10.1161/01.cir.96.4.1102

[28] Whincup PH, Danesh J, Walker M, Lennon L, Thomson A, et al (2002) von Willebrand factor and coronary heart disease: prospective study and meta-analysis. Eur Heart J 23: 1764–1770. 12419296 10.1053/euhj.2001.3237

[29] Keightley AM, Lam YM, Brady JN, Cameron CL, Lillicrap D (1999) Variation at the von Willebrand factor (vWF) gene locus is associated with plasma vWF:Ag levels: identification of three novel single nucleotide polymorphisms in the vWF gene promoter. Blood 93: 4277–4283. 10361125

[30] van der Meer IM, Brouwers GJ, Bulk S, Leebeek FW, van der Kuip DA, et al (2004) Genetic variability of von Willebrand factor and risk of coronary heart disease: the Rotterdam Study. Br J Haematol 124: 343–347. 14717782 10.1046/j.1365-2141.2003.04776.x

[31] Di Bitondo R, Cameron CL, Daly ME, Croft SA, Steeds RP, et al (2001) The-1185 A/G and-1051 G/A dimorphisms in the von Willebrand factor gene promoter and risk of myocardial infarction. Br J Haematol 115: 701–706. 11736957 10.1046/j.1365-2141.2001.03176.x

[32] Gieger C, Radhakrishnan A, Cvejic A, Tang W, Porcu E, et al (2011) New gene functions in megakaryopoiesis and platelet formation. Nature 480: 201–208. 10.1038/nature10659 22139419 PMC3335296

[33] Ruixing Y, Jinzhen W, Shangling P, Weixiong L, Dezhai Y, et al (2008) Sex differences in environmental and genetic factors for hypertension. Am J Med 121: 811–819. 10.1016/j.amjmed.2008.04.026 18724972

[34] Defago MD, Gu D, Hixson JE, Shimmin LC, Rice TK, et al (2013) Common genetic variants in the endothelial system predict blood pressure response to sodium intake: the GenSalt study. Am J Hypertens 26: 643–656. 10.1093/ajh/hps099 23443727 PMC3657485

[35] Yamada Y, Kato K, Hibino T, Yokoi K, Matsuo H, et al (2007) Prediction of genetic risk for metabolic syndrome. Atherosclerosis 191: 298–304. 16806226 10.1016/j.atherosclerosis.2006.05.035

[36] Xu C, Bai R, Zhang D, Li Z, Zhu H, et al (2013) Effects of APOA5–1131T>C (rs662799) on fasting plasma lipids and risk of metabolic syndrome: evidence from a case-control study in China and a meta-analysis. PLoS One 8: e56216 10.1371/journal.pone.0056216 23468858 PMC3585417

[37] Grallert H, Sedlmeier EM, Huth C, Kolz M, Heid IM, et al (2007) APOA5 variants and metabolic syndrome in Caucasians. J Lipid Res 48: 2614–2621. 17768309 10.1194/jlr.M700011-JLR200

[38] Paulik MA, Buckholz RG, Lancaster ME, Dallas WS, Hull-Ryde EA, et al (1998) Development of infrared imaging to measure thermogenesis in cell culture: thermogenic effects of uncoupling protein-2, troglitazone, and beta-adrenoceptor agonists. Pharm Res 15: 944–949. 9647363 10.1023/a:1011993019385

[39] Jia JJ, Tian YB, Cao ZH, Tao LL, Zhang X, et al (2010) The polymorphisms of UCP1 genes associated with fat metabolism, obesity and diabetes. Mol Biol Rep 37: 1513–1522. 10.1007/s11033-009-9550-2 19444646

[40] Dalgaard LT, Pedersen O (2001) Uncoupling proteins: functional characteristics and role in the pathogenesis of obesity and Type II diabetes. Diabetologia 44: 946–965. 11484071 10.1007/s001250100596

[41] Kamatani Y, Matsuda K, Okada Y, Kubo M, Hosono N, et al (2010) Genome-wide association study of hematological and biochemical traits in a Japanese population. Nat Genet 42: 210–215. 10.1038/ng.531 20139978

[42] Pezzolesi MG, Poznik GD, Mychaleckyj JC, Paterson AD, Barati MT, et al (2009) Genome-wide association scan for diabetic nephropathy susceptibility genes in type 1 diabetes. Diabetes 58: 1403–1410. 10.2337/db08-1514 19252134 PMC2682673

[43] Maeda S, Araki S, Babazono T, Toyoda M, Umezono T, et al (2010) Replication study for the association between four loci identified by a genome-wide association study on European American subjects with type 1 diabetes and susceptibility to diabetic nephropathy in Japanese subjects with type 2 diabetes. Diabetes 59: 2075–2079. 10.2337/db10-0067 20460425 PMC2911071

[44] Maric C, Hall JE (2011) Obesity, metabolic syndrome and diabetic nephropathy. Contrib Nephrol 170: 28–35. 10.1159/000324941 21659755 PMC3177237

