# Supplementary material for: Identification of an Interaction between VWF rs7965413 and Platelet Count as a Novel Risk Marker for Metabolic Syndrome: An Extensive Search of Candidate Polymorphisms in a Case-Control Study
Source: PLoS One. 2015 Feb 3;10(2):e0117591. doi: 10.1371/journal.pone.0117591 (PMC4315519; doi:10.1371/journal.pone.0117591)
Supplement: S1 Table — (DOCX) [file pone.0117591.s001.docx]

**Table S1. The 99 genotyped SNPs**

| **SNP** | **Chr** | **Position (GRCh37)** | **Reported gene** | **Other SNP name** | **Reference** |
| --- | --- | --- | --- | --- | --- |
|  |  |  |  |  |  |
| rs1801133 | 1 | 11,856,378 | *MTHFR* | Ala222Val, C677T | Jamison, R. L., et al. (2009) [[1](#_ENREF_1)] |
| rs5063 | 1 | 11,907,648 | *NPPA* | Val7Met, G664A | Rubattu, S., et al. (1999) [[2](#_ENREF_2)] |
| rs1764391 | 1 | 35,260,769 | *GJA4* | Pro319Ser, C1019T | Yamada, Y., et al. (2002) [[3](#_ENREF_3)] |
| rs1137100 | 1 | 66,036,441 | *LEPR* | Lys109Arg | Kilpelainen, T. O., et al. (2008) [[4](#_ENREF_4)] |
| rs267734 | 1 | 150,951,477 | *ANXA9, SETDB1* |  | Kottgen, A., et al. (2010) [[5](#_ENREF_5)] |
| rs6685323 | 1 | 154,295,592 | *AQP10* |  | Ishibashi, K., et al. (2002) [[6](#_ENREF_6)] |
| rs2072660 | 1 | 154,548,721 | *CHRNB2* |  | Pedneault, M., et al. (2014) [[7](#_ENREF_7)] |
| rs1417938 | 1 | 159,684,186 | *CRP* | A1919T | Lange, L. A., et al. (2006) [[8](#_ENREF_8)] |
| rs2073658 | 1 | 161,010,762 | *USF1* |  | Coon, H., et al. (2005) [[9](#_ENREF_9)] |
| rs6131 | 1 | 169,580,885 | *SELP* | Ser290Asn | Tregouet, D. A., et al. (2002) [[10](#_ENREF_10)] |
| rs1061170 | 1 | 196,659,237 | *CFH* | Tyr402His | Kardys, I., et al. (2006) [[11](#_ENREF_11)] |
| rs7539542 | 1 | 202,909,974 | *ADIPOR1* | rs1139646 | Qi, L., et al. (2007) [[12](#_ENREF_12)] |
| rs1539355 | 1 | 202,924,080 | *ADIPOR1* | T–3882C | Ruchat, S. M., et al. (2008) [[13](#_ENREF_13)] |
| rs1800872 | 1 | 206,946,407 | *IL10* | C–592A | Koch, W., et al. (2001) [[14](#_ENREF_14)] |
| rs699 | 1 | 230,845,794 | *AGT* | Met235Thr | Fujiwara, T., et al. (2002) [[15](#_ENREF_15)] |
| rs4762 | 1 | 230,845,977 | *AGT* | Thr174Met | Procopciuc, L. M., et al. (2011) [[16](#_ENREF_16)] |
| rs1260326 | 2 | 27,730,940 | *GCKR* | Leu446Pro | Kottgen, A., et al. (2010) [[5](#_ENREF_5)] |
| rs6720173 | 2 | 44,040,401 | *ABCG5* | Gln604Glu, C1950G | Weggemans, R. M., et al. (2002) [[17](#_ENREF_17)] |
| rs4148217 | 2 | 44,099,433 | *ABCG8* | Thr400Lys, C1199A | Miwa, K., et al. (2005) [[18](#_ENREF_18)] |
| rs2544390 | 2 | 170,204,846 | *LRP2* |  | Hamajima, N., et al. (2012) [[19](#_ENREF_19)] |
| rs3755166 | 2 | 170,219,881 | *LRP2* |  | Hamajima, N., et al. (2012) [[19](#_ENREF_19)] |
| rs696217 | 3 | 10,331,457 | *GHRL* | Leu72Met | Kilpelainen, T. O., et al. (2008) [[4](#_ENREF_4)] |
| rs1175544 | 3 | 12,467,044 | *PPARG* |  | Matsuo, T., et al. (2009) [[20](#_ENREF_20)] |
| rs2228048 | 3 | 30,713,842 | *TGFBR2* | Asn389Asn, C1157T | McKnight, A. J., et al. (2007) [[21](#_ENREF_21)] |
| rs933135 | 3 | 38,052,725 | *PLCD1* | Arg257His, G864A | Nakano, T., et al. (2002) [[22](#_ENREF_22)] |
| rs3732379 | 3 | 39,307,256 | *CX3CR1* | Val249Ile | McDermott, D. H., et al. (2001) [[23](#_ENREF_23)] |
| rs1050450 | 3 | 49,394,834 | *GPX1* | Pro198Leu | Yamada, Y., et al. (2007) [[24](#_ENREF_24)] |
| rs347685 | 3 | 141,807,137 | *TFDP2* |  | Kottgen, A., et al. (2010) [[5](#_ENREF_5)] |
| rs388915 | 3 | 148,447,756 | *AGTR1* | A21942G in intron 2 | Osawa, N., et al. (2007) [[25](#_ENREF_25)] |
| rs5186 | 3 | 148,459,988 | *AGTR1* | A1166C | Kainulainen, K., et al. (1999) [[26](#_ENREF_26)] |
| rs6141 | 3 | 184,090,266 | *THPO* | A5713G | Webb, K. E., et al. (2001) [[27](#_ENREF_27)] |
| rs2241766 | 3 | 186,570,892 | *ADIPOQ* | T45G | Hara, K., et al. (2002) [[28](#_ENREF_28)] |
| rs1501299 | 3 | 186,571,123 | *ADIPOQ* | G276T | Gonzalez-Sanchez, J. L., et al. (2005) [[29](#_ENREF_29)] |
| rs4961 | 4 | 2,906,707 | *ADD1* | Gly460Trp | Cusi, D., et al. (1997) [[30](#_ENREF_30)] |
| rs11722228 | 4 | 9,915,741 | *SLC2A9* |  | Hamajima, N., et al. (2011) [[31](#_ENREF_31)] |
| rs1014290 | 4 | 10,001,861 | *SLC2A9* |  | Hamajima, N., et al. (2011) [[31](#_ENREF_31)] |
| rs8192678 | 4 | 23,815,662 | *PPARGC1A* | Gly482Ser | Andrulionyte, L., et al. (2004) [[32](#_ENREF_32)] |
| rs17319721 | 4 | 77,368,847 | *SHROOM3* |  | Kottgen, A., et al. (2009) [[33](#_ENREF_33)] |
| rs72552713 | 4 | 89,052,957 | *ABCG2* | Q126X | Matsuo, H., et al. (2013) [[34](#_ENREF_34)] |
| rs1229984 | 4 | 100,239,319 | *ADH1B* | Arg47His | Yin, G., et al. (2011) [[35](#_ENREF_35)] |
| rs1800591 | 4 | 100,495,488 | *MTTP* | G–493T | Zak, A., et al. (2008) [[36](#_ENREF_36)] |
| rs1799883 | 4 | 120,241,902 | *FABP2* | Ala54Thr | Vimaleswaran, K. S., et al. (2006) [[37](#_ENREF_37)] |
| rs1800592 | 4 | 141,493,961 | *UCP1* | A–3826G | Kogure, A., et al. (1998) [[38](#_ENREF_38)] |
| rs1801394 | 5 | 7,870,973 | *MTRR* | A66G | Hishida, A., et al. (2013) [[39](#_ENREF_39)] |
| rs11959928 | 5 | 39,397,132 | *DAB2* |  | Kottgen, A., et al. (2010) [[5](#_ENREF_5)] |
| rs2569190 | 5 | 140,012,916 | *CD14* | C260T | Koenig, W., et al. (2002) [[40](#_ENREF_40)] |
| rs6420094 | 5 | 176,817,636 | *SLC34A1* |  | Kottgen, A., et al. (2010) [[5](#_ENREF_5)] |
| rs7756992 | 6 | 20,679,709 | *CDKAL1* |  | Omori, S., et al. (2008) [[41](#_ENREF_41)] |
| rs1041981 | 6 | 31,540,784 | *LTA* | Thr26Asn, C804A | Ozaki, K., et al. (2002) [[42](#_ENREF_42)] |
| rs1800630 | 6 | 31,542,476 | *TNF* | C–863A | Koch, W., et al. (2001) [[14](#_ENREF_14)] |
| rs1008438 | 6 | 31,783,208 | *HSPA1L* | A–110C | Singh, R., et al. (2004) [[43](#_ENREF_43)] |
| rs2070600 | 6 | 32,151,443 | *AGER* | Gly82Ser | Jang, Y., et al. (2007) [[44](#_ENREF_44)] |
| rs2016520 | 6 | 35,378,778 | *PPARD* | T294C | Gouni-Berthold, I., et al. (2005) [[45](#_ENREF_45)] |
| rs881858 | 6 | 43,806,609 | *VEGFA* |  | Kottgen, A., et al. (2010) [[5](#_ENREF_5)] |
| rs1044498 | 6 | 132,172,368 | *ENPP1* | Lys121Gln | Willer, C. J., et al. (2007) [[46](#_ENREF_46)] |
| rs2431260 | 6 | 152,192,331 | *ESR1* |  | Gallagher, C. J., et al. (2007) [[47](#_ENREF_47)] |
| rs8089 | 6 | 169,617,726 | *THBS2* |  | Yamaguchi, S., et al. (2007) [[48](#_ENREF_48)] |
| rs1800796 | 7 | 22,766,246 | *IL6* | G–572C | Hamid, Y. H., et al. (2005) [[49](#_ENREF_49)] |
| rs864745 | 7 | 28,180,556 | *JAZF1* |  | Omori, S., et al. (2009) [[50](#_ENREF_50)] |
| rs1799884 | 7 | 44,229,068 | *GCK* | G–30A | Hishida, A., et al. (2012) [[51](#_ENREF_51)] |
| rs2072183 | 7 | 44,579,180 | *NPC1L1* | Leu272Leu, C1679G | Simon, J. S., et al. (2005) [[52](#_ENREF_52)] |
| rs2070744 | 7 | 150,690,079 | *NOS3* | T–786C | Higashibata, T., et al. (2012) [[53](#_ENREF_53)] |
| rs1799983 | 7 | 150,696,111 | *NOS3* | Glu298Asp | Lembo, G., et al. (2001) [[54](#_ENREF_54)] |
| rs328 | 8 | 19,819,724 | *LPL* | Ser447Ter | Wittrup, H. H., et al. (1999) [[55](#_ENREF_55)] |
| rs10109414 | 8 | 23,751,151 | *STC1* |  | Kottgen, A., et al. (2010) [[5](#_ENREF_5)] |
| rs1346044 | 8 | 31,024,654 | *WRN* | Cys1367Arg | Castro, E., et al. (2000) [[56](#_ENREF_56)] |
| rs2020918 | 8 | 42,072,438 | *PLAT* | C–7351T | Jannes, J., et al. (2004) [[57](#_ENREF_57)] |
| rs4744712 | 9 | 71,434,707 | *PIP5K1B* |  | Kottgen, A., et al. (2010) [[5](#_ENREF_5)] |
| rs501120 | 10 | 44,753,867 | *-* |  | Samani, N. J., et al. (2007) [[58](#_ENREF_58)] |
| rs2237897 | 11 | 2,858,546 | *KCNQ1* |  | Unoki, H., et al. (2008) [[59](#_ENREF_59)] |
| rs5219 | 11 | 17,409,572 | *KCNJ11* | Glu23Lys | Omori, S., et al. (2008) [[41](#_ENREF_41)] |
| rs2075291 | 11 | 116,661,392 | *APOA5* | Gly185Cys, G553T | Hishida, A., et al. (2012) [[51](#_ENREF_51)] |
| rs662799 | 11 | 116,663,707 | *APOA5* | T–1131C | Martinelli, N., et al. (2007) [[60](#_ENREF_60)] |
| rs2854117 | 11 | 116,700,142 | *APOC3* | T–455C | Miller, M., et al. (2007) [[61](#_ENREF_61)] |
| rs12718465 | 11 | 116,707,736 | *APOA1* | Ala61Thr | Nakamura, A., et al. (2013) [[62](#_ENREF_62)] |
| rs11216158 | 11 | 116,711,350 | *APOA1* | XmnI | Mar, R., et al. (2004) [[63](#_ENREF_63)] |
| rs7965413 | 12 | 6,234,889 | *VWF* | G–1051A | Keightley, A. M., et al. (1999) [[64](#_ENREF_64)] |
| rs958812 | 12 | 12,049,662 | *LRP6* |  | Mani, A., et al. (2007) [[65](#_ENREF_65)] |
| rs1799986 | 12 | 57,535,266 | *LRP1* | C766T | Pocathikorn, A., et al. (2003) [[66](#_ENREF_66)] |
| rs3782886 | 12 | 112,110,489 | *BRAP* |  | Ozaki, K., et al. (2009) [[67](#_ENREF_67)] |
| rs1411766 | 13 | 110,252,160 | *-* |  | Maeda, S., et al. (2010) [[68](#_ENREF_68)] |
| rs6046 | 13 | 113,773,159 | *F7* | Arg353Gln | Shimokata, K., et al. (2002) [[69](#_ENREF_69)] |
| rs1255998 | 14 | 64,693,871 | *ESR2* |  | Lo, J. C., et al. (2006) [[70](#_ENREF_70)] |
| rs2467853 | 15 | 45,698,793 | *SPATA5L1* |  | Kottgen, A., et al. (2009) [[33](#_ENREF_33)] |
| rs1800588 | 15 | 58,723,675 | *LIPC* | C–480T | Andersen, R. V., et al. (2003) [[71](#_ENREF_71)] |
| rs6495446 | 15 | 80,154,982 | *MTHFS* |  | Kottgen, A., et al. (2008) [[72](#_ENREF_72)] |
| rs5882 | 16 | 57,016,092 | *CETP* | Ile405Val | Blankenberg, S., et al. (2003) [[73](#_ENREF_73)] |
| rs4673 | 16 | 88,713,236 | *CYBA* | His72Tyr, C242T | Cahilly, C., et al. (2000) [[74](#_ENREF_74)] |
| rs2243093 | 17 | 4,835,895 | *GP1BA* | T–5C | Maguire, J. M., et al. (2008) [[75](#_ENREF_75)] |
| rs1024611 | 17 | 32,579,788 | *CCL2* | G–2578A | Brenner, D., et al. (2006) [[76](#_ENREF_76)] |
| rs2333227 | 17 | 56,358,762 | *MPO* | G–463A | Hoy, A., et al. (2003) [[77](#_ENREF_77)] |
| rs1862513 | 19 | 7,733,793 | *RETN* | C–420G | Osawa, H., et al. (2008) [[78](#_ENREF_78)] |
| rs5498 | 19 | 10,395,683 | *ICAM1* | Glu469Lys | Pola, R., et al. (2003) [[79](#_ENREF_79)] |
| rs1800469 | 19 | 41,860,296 | *TGFB1* | C–509T | Crobu, F., et al. (2008) [[80](#_ENREF_80)] |
| rs405509 | 19 | 45,408,836 | *APOE* | G–219T | Yoshida, T., et al. (2009) [[81](#_ENREF_81)] |
| rs13038305 | 20 | 23,610,262 | *CST3* |  | Gudbjartsson, D. F., et al. (2010) [[82](#_ENREF_82)] |
| rs3918242 | 20 | 44,635,976 | *MMP9* | C–1562T | Zhang, B., et al. (1999) [[83](#_ENREF_83)] |
| rs5629 | 20 | 48,129,706 | *PTGIS* | C1117A | Yamada, Y., et al. (2006) [[84](#_ENREF_84)] |
| rs235326 | 21 | 46,311,813 | *ITGB2* |  | Awaya, T., et al. (2008) [[85](#_ENREF_85)] |

**References**

1. Jamison RL, Shih MC, Humphries DE, Guarino PD, Kaufman JS, et al. (2009) Effect of the MTHFR C677T and A1298C polymorphisms on survival in patients with advanced CKD and ESRD: a prospective study. Am J Kidney Dis 53: 779-789.

2. Rubattu S, Ridker P, Stampfer MJ, Volpe M, Hennekens CH, et al. (1999) The gene encoding atrial natriuretic peptide and the risk of human stroke. Circulation 100: 1722-1726.

3. Yamada Y, Izawa H, Ichihara S, Takatsu F, Ishihara H, et al. (2002) Prediction of the risk of myocardial infarction from polymorphisms in candidate genes. N Engl J Med 347: 1916-1923.

4. Kilpelainen TO, Lakka TA, Laaksonen DE, Mager U, Salopuro T, et al. (2008) Interaction of single nucleotide polymorphisms in ADRB2, ADRB3, TNF, IL6, IGF1R, LIPC, LEPR, and GHRL with physical activity on the risk of type 2 diabetes mellitus and changes in characteristics of the metabolic syndrome: The Finnish Diabetes Prevention Study. Metabolism 57: 428-436.

5. Kottgen A, Pattaro C, Boger CA, Fuchsberger C, Olden M, et al. (2010) New loci associated with kidney function and chronic kidney disease. Nat Genet 42: 376-384.

6. Ishibashi K, Morinaga T, Kuwahara M, Sasaki S, Imai M (2002) Cloning and identification of a new member of water channel (AQP10) as an aquaglyceroporin. Biochim Biophys Acta 1576: 335-340.

7. Pedneault M, Labbe A, Roy-Gagnon MH, Low NC, Dugas E, et al. (2014) The association between CHRN genetic variants and dizziness at first inhalation of cigarette smoke. Addict Behav 39: 316-320.

8. Lange LA, Carlson CS, Hindorff LA, Lange EM, Walston J, et al. (2006) Association of polymorphisms in the CRP gene with circulating C-reactive protein levels and cardiovascular events. JAMA 296: 2703-2711.

9. Coon H, Xin Y, Hopkins PN, Cawthon RM, Hasstedt SJ, et al. (2005) Upstream stimulatory factor 1 associated with familial combined hyperlipidemia, LDL cholesterol, and triglycerides. Hum Genet 117: 444-451.

10. Tregouet DA, Barbaux S, Escolano S, Tahri N, Golmard JL, et al. (2002) Specific haplotypes of the P-selectin gene are associated with myocardial infarction. Hum Mol Genet 11: 2015-2023.

11. Kardys I, Klaver CC, Despriet DD, Bergen AA, Uitterlinden AG, et al. (2006) A common polymorphism in the complement factor H gene is associated with increased risk of myocardial infarction: the Rotterdam Study. J Am Coll Cardiol 47: 1568-1575.

12. Qi L, Doria A, Giorgi E, Hu FB (2007) Variations in adiponectin receptor genes and susceptibility to type 2 diabetes in women: a tagging-single nucleotide polymorphism haplotype analysis. Diabetes 56: 1586-1591.

13. Ruchat SM, Loos RJ, Rankinen T, Vohl MC, Weisnagel SJ, et al. (2008) Associations between glucose tolerance, insulin sensitivity and insulin secretion phenotypes and polymorphisms in adiponectin and adiponectin receptor genes in the Quebec Family Study. Diabet Med 25: 400-406.

14. Koch W, Kastrati A, Bottiger C, Mehilli J, von Beckerath N, et al. (2001) Interleukin-10 and tumor necrosis factor gene polymorphisms and risk of coronary artery disease and myocardial infarction. Atherosclerosis 159: 137-144.

15. Fujiwara T, Katsuya T, Matsubara M, Mikami T, Ishikawa K, et al. (2002) T+31C polymorphism of angiotensinogen gene and nocturnal blood pressure decline: the Ohasama study. Am J Hypertens 15: 628-632.

16. Procopciuc LM, Caracostea G, Zaharie G, Puscas M, Iordache G, et al. (2011) Maternal/newborn genotype contribution of the renin-angiotensin system (Met235Thr, Thr174Met, I/D-ACE, A2350G-ACE, A1166C-AT2R1, C3123A- AT2R2, 83A/G-REN) to the risk of pre-eclampsia: a Romanian study. J Renin Angiotensin Aldosterone Syst 12: 539-548.

17. Weggemans RM, Zock PL, Tai ES, Ordovas JM, Molhuizen HO, et al. (2002) ATP binding cassette G5 C1950G polymorphism may affect blood cholesterol concentrations in humans. Clin Genet 62: 226-229.

18. Miwa K, Inazu A, Kobayashi J, Higashikata T, Nohara A, et al. (2005) ATP-binding cassette transporter G8 M429V polymorphism as a novel genetic marker of higher cholesterol absorption in hypercholesterolaemic Japanese subjects. Clin Sci (Lond) 109: 183-188.

19. Hamajima N, Naito M, Okada R, Kawai S, Yin G, et al. (2012) Significant interaction between LRP2 rs2544390 in intron 1 and alcohol drinking for serum uric acid levels among a Japanese population. Gene 503: 131-136.

20. Matsuo T, Nakata Y, Katayama Y, Iemitsu M, Maeda S, et al. (2009) PPARG genotype accounts for part of individual variation in body weight reduction in response to calorie restriction. Obesity 17: 1924-1931.

21. McKnight AJ, Savage DA, Patterson CC, Sadlier D, Maxwell AP (2007) Resequencing of genes for transforming growth factor beta1 (TGFB1) type 1 and 2 receptors (TGFBR1, TGFBR2), and association analysis of variants with diabetic nephropathy. BMC Med Genet 8: 5.

22. Nakano T, Osanai T, Tomita H, Sekimata M, Homma Y, et al. (2002) Enhanced activity of variant phospholipase C-delta1 protein (R257H) detected in patients with coronary artery spasm. Circulation 105: 2024-2029.

23. McDermott DH, Halcox JP, Schenke WH, Waclawiw MA, Merrell MN, et al. (2001) Association between polymorphism in the chemokine receptor CX3CR1 and coronary vascular endothelial dysfunction and atherosclerosis. Circ Res 89: 401-407.

24. Yamada Y, Ando F, Shimokata H (2007) Association of gene polymorphisms with blood pressure and the prevalence of hypertension in community-dwelling Japanese individuals. Int J Mol Med 19: 675-683.

25. Osawa N, Koya D, Araki S, Uzu T, Tsunoda T, et al. (2007) Combinational effect of genes for the renin-angiotensin system in conferring susceptibility to diabetic nephropathy. J Hum Genet 52: 143-151.

26. Kainulainen K, Perola M, Terwilliger J, Kaprio J, Koskenvuo M, et al. (1999) Evidence for involvement of the type 1 angiotensin II receptor locus in essential hypertension. Hypertension 33: 844-849.

27. Webb KE, Martin JF, Hamsten A, Eriksson P, Iacoviello L, et al. (2001) Polymorphisms in the thrombopoietin gene are associated with risk of myocardial infarction at a young age. Atherosclerosis 154: 703-711.

28. Hara K, Boutin P, Mori Y, Tobe K, Dina C, et al. (2002) Genetic variation in the gene encoding adiponectin is associated with an increased risk of type 2 diabetes in the Japanese population. Diabetes 51: 536-540.

29. Gonzalez-Sanchez JL, Zabena CA, Martinez-Larrad MT, Fernandez-Perez C, Perez-Barba M, et al. (2005) An SNP in the adiponectin gene is associated with decreased serum adiponectin levels and risk for impaired glucose tolerance. Obes Res 13: 807-812.

30. Cusi D, Barlassina C, Azzani T, Casari G, Citterio L, et al. (1997) Polymorphisms of alpha-adducin and salt sensitivity in patients with essential hypertension. Lancet 349: 1353-1357.

31. Hamajima N, Okada R, Kawai S, Hishida A, Morita E, et al. (2011) Significant association of serum uric acid levels with SLC2A9 rs11722228 among a Japanese population. Mol Genet Metab 103: 378-382.

32. Andrulionyte L, Zacharova J, Chiasson JL, Laakso M, Group S-NS (2004) Common polymorphisms of the PPAR-gamma2 (Pro12Ala) and PGC-1alpha (Gly482Ser) genes are associated with the conversion from impaired glucose tolerance to type 2 diabetes in the STOP-NIDDM trial. Diabetologia 47: 2176-2184.

33. Kottgen A, Glazer NL, Dehghan A, Hwang SJ, Katz R, et al. (2009) Multiple loci associated with indices of renal function and chronic kidney disease. Nat Genet 41: 712-717.

34. Matsuo H, Ichida K, Takada T, Nakayama A, Nakashima H, et al. (2013) Common dysfunctional variants in ABCG2 are a major cause of early-onset gout. Sci Rep 3: 2014.

35. Yin G, Hamajima N, Morita M, Tajima O, Tabata S, et al. (2011) Lack of influence of the ADH1B Arg47His genetic polymorphism on risk of colorectal adenoma in middle-aged Japanese men. Asian Pac J Cancer Prev 12: 297-302.

36. Zak A, Jachymova M, Tvrzicka E, Vecka M, Duffkova L, et al. (2008) The influence of polymorphism of -493G/T MTP gene promoter and metabolic syndrome on lipids, fatty acids and oxidative stress. J Nutr Biochem 19: 634-641.

37. Vimaleswaran KS, Radha V, Mohan V (2006) Thr54 allele carriers of the Ala54Thr variant of FABP2 gene have associations with metabolic syndrome and hypertriglyceridemia in urban South Indians. Metabolism 55: 1222-1226.

38. Kogure A, Yoshida T, Sakane N, Umekawa T, Takakura Y, et al. (1998) Synergic effect of polymorphisms in uncoupling protein 1 and beta3-adrenergic receptor genes on weight loss in obese Japanese. Diabetologia 41: 1399.

39. Hishida A, Okada R, Guang Y, Naito M, Wakai K, et al. (2013) MTHFR, MTR and MTRR polymorphisms and risk of chronic kidney disease in Japanese: cross-sectional data from the J-MICC Study. Int Urol Nephrol 45: 1613-1620.

40. Koenig W, Khuseyinova N, Hoffmann MM, Marz W, Frohlich M, et al. (2002) CD14 C(-260)-->T polymorphism, plasma levels of the soluble endotoxin receptor CD14, their association with chronic infections and risk of stable coronary artery disease. J Am Coll Cardiol 40: 34-42.

41. Omori S, Tanaka Y, Takahashi A, Hirose H, Kashiwagi A, et al. (2008) Association of CDKAL1, IGF2BP2, CDKN2A/B, HHEX, SLC30A8, and KCNJ11 with susceptibility to type 2 diabetes in a Japanese population. Diabetes 57: 791-795.

42. Ozaki K, Ohnishi Y, Iida A, Sekine A, Yamada R, et al. (2002) Functional SNPs in the lymphotoxin-alpha gene that are associated with susceptibility to myocardial infarction. Nat Genet 32: 650-654.

43. Singh R, Kolvraa S, Bross P, Gregersen N, Andersen Nexo B, et al. (2004) Association between low self-rated health and heterozygosity for -110A > C polymorphism in the promoter region of HSP70-1 in aged Danish twins. Biogerontology 5: 169-176.

44. Jang Y, Kim JY, Kang SM, Kim JS, Chae JS, et al. (2007) Association of the Gly82Ser polymorphism in the receptor for advanced glycation end products (RAGE) gene with circulating levels of soluble RAGE and inflammatory markers in nondiabetic and nonobese Koreans. Metabolism 56: 199-205.

45. Gouni-Berthold I, Giannakidou E, Faust M, Berthold HK, Krone W (2005) The peroxisome proliferator-activated receptor delta +294T/C polymorphism in relation to lipoprotein metabolism in patients with diabetes mellitus type 2 and in non-diabetic controls. Atherosclerosis 183: 336-341.

46. Willer CJ, Bonnycastle LL, Conneely KN, Duren WL, Jackson AU, et al. (2007) Screening of 134 single nucleotide polymorphisms (SNPs) previously associated with type 2 diabetes replicates association with 12 SNPs in nine genes. Diabetes 56: 256-264.

47. Gallagher CJ, Langefeld CD, Gordon CJ, Campbell JK, Mychaleckyj JC, et al. (2007) Association of the estrogen receptor-alpha gene with the metabolic syndrome and its component traits in African-American families: the Insulin Resistance Atherosclerosis Family Study. Diabetes 56: 2135-2141.

48. Yamaguchi S, Yamada Y, Matsuo H, Segawa T, Watanabe S, et al. (2007) Gender differences in the association of gene polymorphisms with type 2 diabetes mellitus. Int J Mol Med 19: 631-637.

49. Hamid YH, Rose CS, Urhammer SA, Glumer C, Nolsoe R, et al. (2005) Variations of the interleukin-6 promoter are associated with features of the metabolic syndrome in Caucasian Danes. Diabetologia 48: 251-260.

50. Omori S, Tanaka Y, Horikoshi M, Takahashi A, Hara K, et al. (2009) Replication study for the association of new meta-analysis-derived risk loci with susceptibility to type 2 diabetes in 6,244 Japanese individuals. Diabetologia 52: 1554-1560.

51. Hishida A, Morita E, Naito M, Okada R, Wakai K, et al. (2012) Associations of apolipoprotein A5 (APOA5), glucokinase (GCK) and glucokinase regulatory protein (GCKR) polymorphisms and lifestyle factors with the risk of dyslipidemia and dysglycemia in Japanese - a cross-sectional data from the J-MICC Study. Endocr J 59: 589-599.

52. Simon JS, Karnoub MC, Devlin DJ, Arreaza MG, Qiu P, et al. (2005) Sequence variation in NPC1L1 and association with improved LDL-cholesterol lowering in response to ezetimibe treatment. Genomics 86: 648-656.

53. Higashibata T, Hamajima N, Naito M, Kawai S, Yin G, et al. (2012) eNOS genotype modifies the effect of leisure-time physical activity on serum triglyceride levels in a Japanese population. Lipids Health Dis 11: 150.

54. Lembo G, De Luca N, Battagli C, Iovino G, Aretini A, et al. (2001) A common variant of endothelial nitric oxide synthase (Glu298Asp) is an independent risk factor for carotid atherosclerosis. Stroke 32: 735-740.

55. Wittrup HH, Tybjaerg-Hansen A, Nordestgaard BG (1999) Lipoprotein lipase mutations, plasma lipids and lipoproteins, and risk of ischemic heart disease. A meta-analysis. Circulation 99: 2901-2907.

56. Castro E, Edland SD, Lee L, Ogburn CE, Deeb SS, et al. (2000) Polymorphisms at the Werner locus: II. 1074Leu/Phe, 1367Cys/Arg, longevity, and atherosclerosis. Am J Med Genet 95: 374-380.

57. Jannes J, Hamilton-Bruce MA, Pilotto L, Smith BJ, Mullighan CG, et al. (2004) Tissue plasminogen activator -7351C/T enhancer polymorphism is a risk factor for lacunar stroke. Stroke 35: 1090-1094.

58. Samani NJ, Erdmann J, Hall AS, Hengstenberg C, Mangino M, et al. (2007) Genomewide association analysis of coronary artery disease. N Engl J Med 357: 443-453.

59. Unoki H, Takahashi A, Kawaguchi T, Hara K, Horikoshi M, et al. (2008) SNPs in KCNQ1 are associated with susceptibility to type 2 diabetes in East Asian and European populations. Nat Genet 40: 1098-1102.

60. Martinelli N, Trabetti E, Bassi A, Girelli D, Friso S, et al. (2007) The -1131 T>C and S19W APOA5 gene polymorphisms are associated with high levels of triglycerides and apolipoprotein C-III, but not with coronary artery disease: an angiographic study. Atherosclerosis 191: 409-417.

61. Miller M, Rhyne J, Chen H, Beach V, Ericson R, et al. (2007) APOC3 promoter polymorphisms C-482T and T-455C are associated with the metabolic syndrome. Arch Med Res 38: 444-451.

62. Nakamura A, Niimura H, Kuwabara K, Takezaki T, Morita E, et al. (2013) Gene-Gene Combination Effect and Interactions among ABCA1, APOA1, SR-B1, and CETP Polymorphisms for Serum High-Density Lipoprotein-Cholesterol in the Japanese Population. PLoS One 8: e82046.

63. Mar R, Pajukanta P, Allayee H, Groenendijk M, Dallinga-Thie G, et al. (2004) Association of the APOLIPOPROTEIN A1/C3/A4/A5 gene cluster with triglyceride levels and LDL particle size in familial combined hyperlipidemia. Circ Res 94: 993-999.

64. Keightley AM, Lam YM, Brady JN, Cameron CL, Lillicrap D (1999) Variation at the von Willebrand factor (vWF) gene locus is associated with plasma vWF:Ag levels: identification of three novel single nucleotide polymorphisms in the vWF gene promoter. Blood 93: 4277-4283.

65. Mani A, Radhakrishnan J, Wang H, Mani A, Mani MA, et al. (2007) LRP6 mutation in a family with early coronary disease and metabolic risk factors. Science 315: 1278-1282.

66. Pocathikorn A, Granath B, Thiry E, Van Leuven F, Taylor R, et al. (2003) Influence of exonic polymorphisms in the gene for LDL receptor-related protein (LRP) on risk of coronary artery disease. Atherosclerosis 168: 115-121.

67. Ozaki K, Sato H, Inoue K, Tsunoda T, Sakata Y, et al. (2009) SNPs in BRAP associated with risk of myocardial infarction in Asian populations. Nat Genet 41: 329-333.

68. Maeda S, Araki S, Babazono T, Toyoda M, Umezono T, et al. (2010) Replication study for the association between four Loci identified by a genome-wide association study on European American subjects with type 1 diabetes and susceptibility to diabetic nephropathy in Japanese subjects with type 2 diabetes. Diabetes 59: 2075-2079.

69. Shimokata K, Kondo T, Ohno M, Takeshita K, Inden Y, et al. (2002) Effects of coagulation Factor VII polymorphisms on the coronary artery disease in Japanese: Factor VII polymorphism and coronary disease. Thromb Res 105: 493-498.

70. Lo JC, Zhao X, Scuteri A, Brockwell S, Sowers MR (2006) The association of genetic polymorphisms in sex hormone biosynthesis and action with insulin sensitivity and diabetes mellitus in women at midlife. Am J Med 119: S69-78.

71. Andersen RV, Wittrup HH, Tybjaerg-Hansen A, Steffensen R, Schnohr P, et al. (2003) Hepatic lipase mutations,elevated high-density lipoprotein cholesterol, and increased risk of ischemic heart disease: the Copenhagen City Heart Study. J Am Coll Cardiol 41: 1972-1982.

72. Kottgen A, Kao WH, Hwang SJ, Boerwinkle E, Yang Q, et al. (2008) Genome-wide association study for renal traits in the Framingham Heart and Atherosclerosis Risk in Communities Studies. BMC Med Genet 9: 49.

73. Blankenberg S, Rupprecht HJ, Bickel C, Jiang XC, Poirier O, et al. (2003) Common genetic variation of the cholesteryl ester transfer protein gene strongly predicts future cardiovascular death in patients with coronary artery disease. J Am Coll Cardiol 41: 1983-1989.

74. Cahilly C, Ballantyne CM, Lim DS, Gotto A, Marian AJ (2000) A variant of p22(phox), involved in generation of reactive oxygen species in the vessel wall, is associated with progression of coronary atherosclerosis. Circ Res 86: 391-395.

75. Maguire JM, Thakkinstian A, Sturm J, Levi C, Lincz L, et al. (2008) Polymorphisms in platelet glycoprotein 1balpha and factor VII and risk of ischemic stroke: a meta-analysis. Stroke 39: 1710-1716.

76. Brenner D, Labreuche J, Touboul PJ, Schmidt-Petersen K, Poirier O, et al. (2006) Cytokine polymorphisms associated with carotid intima-media thickness in stroke patients. Stroke 37: 1691-1696.

77. Hoy A, Leininger-Muller B, Poirier O, Siest G, Gautier M, et al. (2003) Myeloperoxidase polymorphisms in brain infarction. Association with infarct size and functional outcome. Atherosclerosis 167: 223-230.

78. Osawa H, Ochi M, Tabara Y, Kato K, Yamauchi J, et al. (2008) Serum resistin is positively correlated with the accumulation of metabolic syndrome factors in type 2 diabetes. Clin Endocrinol (Oxf) 69: 74-80.

79. Pola R, Flex A, Gaetani E, Flore R, Serricchio M, et al. (2003) Synergistic effect of -174 G/C polymorphism of the interleukin-6 gene promoter and 469 E/K polymorphism of the intercellular adhesion molecule-1 gene in Italian patients with history of ischemic stroke. Stroke 34: 881-885.

80. Crobu F, Palumbo L, Franco E, Bergerone S, Carturan S, et al. (2008) Role of TGF-beta1 haplotypes in the occurrence of myocardial infarction in young Italian patients. BMC Med Genet 9: 13.

81. Yoshida T, Kato K, Fujimaki T, Yokoi K, Oguri M, et al. (2009) Association of a polymorphism of the apolipoprotein E gene with chronic kidney disease in Japanese individuals with metabolic syndrome. Genomics 93: 221-226.

82. Gudbjartsson DF, Holm H, Indridason OS, Thorleifsson G, Edvardsson V, et al. (2010) Association of variants at UMOD with chronic kidney disease and kidney stones-role of age and comorbid diseases. PLoS Genet 6: e1001039.

83. Zhang B, Ye S, Herrmann SM, Eriksson P, de Maat M, et al. (1999) Functional polymorphism in the regulatory region of gelatinase B gene in relation to severity of coronary atherosclerosis. Circulation 99: 1788-1794.

84. Yamada Y, Matsuo H, Segawa T, Watanabe S, Kato K, et al. (2006) Assessment of the genetic component of hypertension. Am J Hypertens 19: 1158-1165.

85. Awaya T, Yokosaki Y, Yamane K, Usui H, Kohno N, et al. (2008) Gene-environment association of an ITGB2 sequence variant with obesity in ethnic Japanese. Obesity 16: 1463-1466.
